# Competence development among healthcare professionals through an online diversity training - a scoping review

**DOI:** 10.1186/s12909-025-07745-z

**Published:** 2025-08-26

**Authors:** Christian Kempny, Tugba Aksakal, Yüce Yilmaz-Aslan, Patrick Brzoska

**Affiliations:** Faculty of Health, School of Medicine, Health Services Research, Witten/Herdecke University, Witten, Germany

**Keywords:** Online diversity training, Competence development, Scoping review, Training duration, Training methods, Healthcare professionals

## Abstract

**Background:**

The current shortage of healthcare professionals makes international recruitment necessary. This also increases the diversity of teams in terms of characteristics such as age, gender, profession, values, and attitudes. The increase in team diversity can lead to challenges. Online diversity training may improve collaboration within diverse teams and facilitate integration. However, there is limited empirical evidence on the effectiveness of different methods of online diversity training for healthcare professionals. This scoping review aims to map existing research in the field.

**Methods:**

The search was conducted in PubMed, Business Source Premier, CINAHL and PsycInfo. Empirical studies published in English and German between 2014 and 2024 that evaluated online or hybrid diversity training were included.

**Results:**

Of the 4,110 studies identified, 23 met the inclusion criteria. Most were conducted in the United States (14 studies). The duration of training sessions varied from one hour to several weeks, with a predominant thematic focus on cultural diversity (16 studies). Program formats also varied, ranging from live online lectures to digital training courses delivered through learning portals or virtual reality (VR) simulations. Results demonstrated that online training has the potential to enhance diversity competence. Participants reported increased self-efficacy and knowledge, as well as a greater willingness to confront biases. Some studies have also observed changes in participants’ behavior post-training.

**Conclusion:**

Online diversity training programs are promising for enhancing cultural competence among healthcare professionals, particularly in knowledge and attitudes. However, technical and organizational challenges may hinder implementation, and further research is needed to clarify their effectiveness in achieving long-term behavioral change.

## Background

The current shortage of healthcare professionals underscores the need for international recruitment [1]. Recruiting staff from abroad increases the diversity of teams in terms of characteristics such as age, gender, profession, values, and attitudes. This can be challenging for all stakeholders involved. Discrimination and stigmatization [2–4], inequality of opportunity [3, 5], and exclusion [6, 7] are common problems encountered in diverse teams, especially when adequate resources for collaboration are lacking. Language barriers [8] and communication difficulties [3, 8, 9] may also be more pronounced. Addressing these challenges, support programs tailored to diverse teams have shown potential to improve job satisfaction [10], team performance [11, 12], retention [6, 13], and the quality of care delivered [9, 10, 12, 14]. Such programs include diversity training [12, 15, 16], diversity leadership [7, 11], and mentoring programs [17].

Of particular relevance is diversity competence, which encompasses a range of skills and knowledge. In research on intercultural competence, the literature has identified three key dimensions: cognitive, affective, and behavioral. The cognitive level of diversity competence includes knowledge of different cultures or the particularities of LGBTQ + patients. The affective level encompasses attitudes toward minority groups, motivation to address minority issues, and sensitivity. Finally, the behavioral level addresses the skills and abilities required to work with diverse populations effectively [18–20].

Promoting diversity competence through training is crucial for healthcare providers to develop practical communication skills and foster positive internal attitudes. The primary aim of these trainings is to increase awareness and understanding of diverse policies, thereby reducing stereotypes, prejudice, and discrimination [21]. Additionally, diversity training fosters better intergroup relationships [21]. The effectiveness of diversity training has already been proven across several induestries [22, 23, 24], instilling confidence in its potential to improve team dynamics within the healthcare sector as well. Such training might increase healthcare workers’ confidence in managing diversity.

There are different modes in which such training can be delivered. One key decision involves whether content is to be delivered in a classroom setting with a teacher (in person or online) or in an online environment without a teacher. Time-consuming face-to-face training courses (be it in person or online) are often challenging to implement, as the effort and organization involved may prevent the participation of already busy staff [25]. As an alternative to face-to-face training, online learning environments (e-learning) have been developed. For healthcare professionals, such online trainings help to promote diverse skills in the shortest possible timeframe, thereby avoiding additional time pressures on staff, especially when compared with long face-to-face seminars or online lectures [26].

One shortcoming inherent to many currently available online trainings is that they frequently employ standardized training courses designed and structured equally for all learners [27]. These learning environments constitute nonadaptive e-learning environments (NEEs) in which all users access the same content in the same order [27]. NEEs represent a one-size-fits-all solution that does not consider learners’ differing levels of knowledge or whether particular learning strategies may be more effective for different learners. In contrast, adaptive online trainings also exist, providing a flexible learning experience that can be easily tailored to users’ needs and schedules. Despite a growth in the implementation and usage of different types of online training, there is limited evidence on the efficacy of these training methods and their value for participants. In particular, the impact of training duration on effectiveness remains unclear.

Considering these limitations, the present scoping review aims to map the research completed in the field systematically and to identify gaps in current knowledge regarding the effectiveness of online diversity training. The following research question was formulated: What is the current state of knowledge regarding competence development in online diversity training, specifically in terms of training duration and methods? The scoping review is conducted as part of a project aiming to develop a diversity training program for rehabilitation facilities in Germany.

## Methods

This scoping review was conducted using the six-stage methodological framework proposed by Arksey and O’Malley [28] and reported following the PRISMA-ScR guidelines.

Studies published between 2014 and 2024 were included in the review, considering that ten years represent a significant period for digital training. Studies had to be published in either German or English, involving either online or hybrid diversity training. The content of the diversity training should make up at least 75% of the total training. Additionally, training sessions could have been delivered synchronously or asynchronously. Studies were excluded if more than 50% of participants were students or school pupils. Furthermore, any training programs that lacked an analysis of effectiveness, whether qualitative or quantitative, were also excluded from consideration (Table 1).

**Table 1:** Inclusion and exclusion criteria

Inclusion Criteria	Exclusion Criteria
Year of publication: 2014–2024	More than 50% of the study population are students or pupils.
Language: German or English	Training descriptions without any effectiveness analysis (qualitative or quantitative)
Training conducted online or in a hybrid format	
Training is conducted synchronously or asynchronously.	

**Fig. 1:**
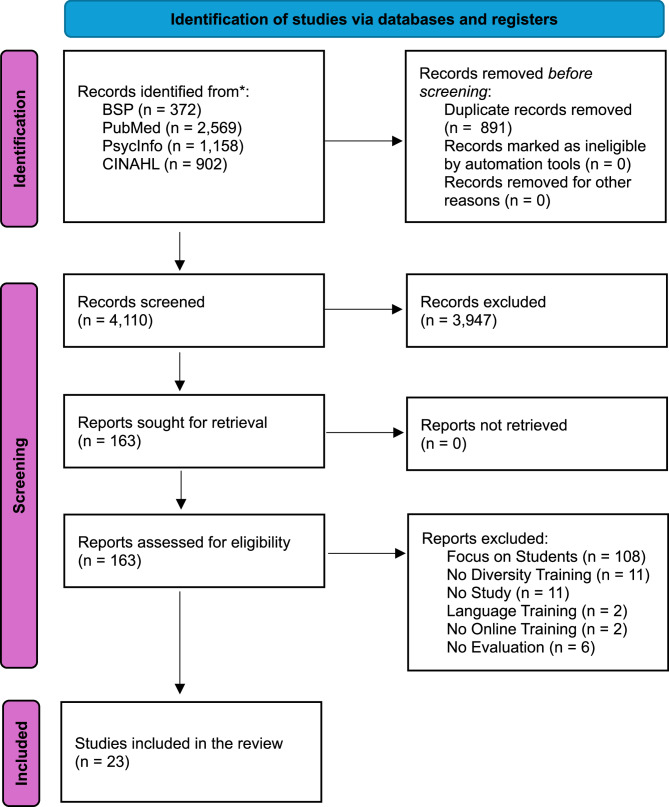
PRISMA flow diagram

To identify potentially relevant documents, the following bibliographic databases were searched for the time frame of January 2014 to April 2024: Business Source Premier, PubMed, PsycINFO, and CINAHL. An experienced research team drafted and refined the search strategies via regular team discussions. The final search results were exported into Rayyan, and duplicates were removed via the Rayyan Duplicate Finder tool. This scoping review was conducted in accordance with the PRISMA-ScR guidelines (Fig. 1).

The search strategy, which comprises three main components (digital format, training, and diversity) and additional filters for publication year and language, is detailed in Table 2.

**Table 2:** Overview of the search strategy, including search components, search terms, and applied filters

Component	Search Terms
Digital Format	online OR “e-learning” OR “virtual*” OR “web-basiert” OR “web-based” OR “digitale plattformen” OR “internet-basiert” OR “cyber training” OR “virtual classroom” OR “digital learning” OR “digital”
Training	training OR scheduling OR weiterbildung OR “fortbildung” OR “lehrprogramm*” OR “bildungsprogramm*” OR “education” OR “training program*” OR “professional development” OR “skill development”
Diversity	diversität OR “kulturelle kompetenz” OR inklusion OR inclusion OR “soziale gerechtigkeit” OR “interkulturelle kompetenz” OR “vielfaltstraining” OR “anti-bias-training” OR “diversity” OR “cultural competence” OR “inclusivity” OR “social justice” OR “intercultural” OR “equity” OR “anti-discrimination”
Filters applied	Year: 2014–2024 Language: German, English

All records were first imported into Rayyan, where duplicate references were automatically identified and removed. Two reviewers (CK and TA) then reviewed all titles and abstracts in Rayyan and discussed studies without consensus in the screening process. In cases of disagreement, the study proceeded to the next step, full paper screening, to prevent the possible exclusion of relevant research. The title and abstract screening and screening manual were discussed and consented by all researchers working on this study (CK, TA, YA, PB). CK designed the data extraction manual, which was then also discussed and consented by the research team.

Two reviewers (CK and TA) jointly developed the data-charting form to determine which variables to extract. One reviewer (CK) charted the data, discussed the results, and continuously updated the form in an iterative process.

We abstracted data on article characteristics (e.g., country of origin, participants, methods, intervention groups), engagement characteristics (e.g., training methods), contextual characteristics (e.g., factors, training content, assessment scales, diversity dimension trained), and the results of online diversity training (e.g., knowledge, attitudes, behavior).

Regardless of quality, all studies meeting these prerequisites were included in the final scoping review. This means that studies containing methodological errors and inaccuracies in their method descriptions were also initially included. The studies were assessed as part of our analysis. In doing so, attention was given to whether methodologically flawed studies yielded different results from studies that were considered methodologically sound.

We grouped studies based on training duration and training methods (synchronous, asynchronous, simulation). The categorization of training duration into “up to 1 hour,” “1–8 hours,” and “more than 8 hours” was based on the range and frequency of durations reported in the included studies. As there is no universally established standard for defining short-, medium-, and long-term interventions in the context of online diversity training, this pragmatic approach enabled meaningful grouping and facilitated comparative analysis. We analyzed and summarized their content based on the three dimensions of diversity competence: cognitive, affective, and behavioral. We also collected information about the general effectiveness of the training provided. No formal synthesis, such as thematic mapping or effect size summary, was conducted due to the substantial methodological and content-related heterogeneity across the included studies. The diversity in study designs, intervention types, target groups, and outcome measures did not allow for a meaningful quantitative or highly structured qualitative synthesis. Instead, we summarized the findings descriptively and narratively, as is recommended for scoping reviews.

## Results

Table 3 provides an overview of each study included in the scoping review. For each study, the table lists the year of publication, number of participants, country of the study, method, study group, types of diversity, and duration of the training.

**Table 3:** Characteristics, results, and measures of the individual studies included in the scoping review

Ref.	Year	Country	Method	Study group	*N*	Diversity dimension	Duration	Effect	Measures
[29]	2024	Germany	Mixed-methods	Mental health professionals	173	Transcultural competence	6 weeks	− Awareness and participation improved. − No improvement in transcultural challenges.	Self-report (transcultural competence (OnTracc); training satisfaction)
[30]	2021	South Korea	Mixed-methods	Healthcare professionals	42	Cultural competence	7 × 15–30 min	− No effect identified.	Self report (feasibility (EL-CCP); preliminary efficacy (CCSN-SF); organizational cultural competence measure for human service agencies; migrant trust and satisfaction)
[31]	2019	USA	Quantitative	Employee	3016	Gender and race	68 min	− Training boosted support for women. − Participants recognized their own gender biases more and demonstrated improved behavior. − Positive effects on gender-inclusive intentions. − Spillover effects from gender bias training on racial bias topics.	Self-report (attitudinal support for women; gender bias acknowledgment; racial bias acknowledgment; gender-inclusive intentions)
[32]	2018	USA	Quantitative	Healthcare professionals	1745	Cultural competence	4 × 5–15 min	− Most participants recognized that minorities experience bias in care, which affects decisions and stereotypes. − That clinical experience, religion, and spirituality influence responses to illness was acknowledged.	Self-reported skills (bias, stereotype, diet, religion)
[33]	2023	USA and Pakistan	Mixed-methods	Healthcare professionals	29	Cultural competence and sensitivity	6 weeks	− Participants found the training valuable. − Pakistani and US doctors improved intercultural care and saw cultural competence training as necessary.	Self-report (Clinical Cultural Competency Questionnaire (CCCQ))
[34]	2020	USA	Quantitative	Employee	433	Belief generation and cross-cultural concepts	Few minutes	− All groups improved through cultural interactions. − Belief generation enhanced cultural learning.	Measures inside a simulation (process data, belief entries, feedback analysis) and a performance quiz
[35]	2022	USA	Quantitative	Healthcare professionals	158	Prejudices	Few minutes	− Participation in the simulation reduced negative emotions and attitudes post-training. − A single simulation proved insufficient in changing patient attitudes or behavior.	Self-report with non-validated scales (emotional reactions, expectations, attribution error, internal/external motivation to respond without prejudice)
[36]	2022	USA	Quantitative	Healthcare professionals	40	Transgender and nonbinary	28 × 60–90 min	− Genetic counseling self-efficacy and knowledge improved significantly. − Participants reduced the gender-affirmation knowledge gap. − The online program was well-received.	Multiple-choice questions (comfort working with TGNB patients; impact of education on knowledge; clinical self-efficacy)
[37]	2019	Australia	Quantitative	Healthcare professionals	53	Cultural competence	4 × 30 min	− Online tools with short interventions were considered helpful. − Participants gained new skills for minority patient care. − Participants described effectiveness as helpful.	Self-report with partly non-validated scales (experience of using and satisfaction with the online program; self-rated competence in communicating with minority patients; practices and attitudes while interacting with people with limited English proficiency; relative responsibility of health professionals and hospitals to adapt to the needs of people from minority backgrounds)
[38]	2022	USA	Quantitative	Mental health professionals	121	LGBTQ	11 weeks	− Participants improved in LGBTQ competence, cognitive behavioral therapy (CBT) knowledge, skills familiarity, and use. − Training did not affect LGBTQ cultural humility.	Self-reports and knowledge tests (Sexual Orientation Counselor Competency Scale (SOCCS) – skills subscale; multidimensional cultural humility scale (MCHS); minority stress knowledge; CBT/LGBTQ-affirmative CBT knowledge; familiarity with and use of LGBTQ-affirmative CBT skills; LGBTQ-affirmative CBT skills measured through simulated practice)
[39]	2021	USA	Quantitative	Healthcare professionals	192	Disability competent care	1 h	− Following the training, participants engaged in more meaningful, patient-centered actions. − Shift toward the social model of disability, focusing on the care environment and treatment barriers.	Quantitative assessment (conceptualizations of disability; action steps to facilitate disability-competent care; training effectiveness; self-assessed knowledge gain)
[40]	2022	Iran	Quantitative	Nurse educators	65	Cultural competence	3 × 2 h	− Participants in the intervention improved their cultural competence. − Competence increased in all areas: awareness, knowledge, skills, encounters, desire, and teaching.	Self-report (Cultural Diversity Questionnaire for Nurse Educators (CDQNE); Cultural Diversity Questionnaire for Nurse Educators revised (CDQNE-R); Transcultural Teaching Behaviors (TTB))
[41]	2021	Israel	Quantitative	Healthcare professionals	154	Cultural competence	30 min	− Participants found training helpful and improved cultural competence in encounters, knowledge, skills, and attitudes.	Self-report (Clinical Cultural Competency Questionnaire (CCCQ) with adaptation)
[42]	2021	USA	Qualitative	Teacher	36	Cultural awareness	3 meetings	− Collaborative teaching improved analysis methods. − The intervention offered practical experience teaching bilingual students online and on-site. − Helped teachers develop skills in dealing with diverse students.	Qualitative analysis
[43]	2022	USA	Quantitative	Mental health professionals	523	Intercultural competence	3 × 90 min	− Awareness and knowledge increased with each webinar and remained steady after two weeks. − White participants experienced the most considerable increase in awareness after the first webinar.	Self-report (knowledge scale for cross-cultural psychiatric training; professionals’ awareness of competencies for supporting)
[44]	2020	USA	Quantitative	Mental health professionals	11	Intercultural competence	6 weeks	− Participants improved their cultural competencies.	Self-report (Clinical Cultural Competence Questionnaire (CCCQ) with adaptation)
[45]	2018	USA	Quantitative	Healthcare professionals	26	Cultural Competence and LGBTQ	1 h	− No effects.	Self-report (LGB knowledge and attitudes (LGB-KASH); AttitudesTAoward Transgender Individuals Scale (ATTIS); LGBT skills; LGBT knowledge; social desirability)
[46]	2021	USA	Qualitative	Faculty member	21	Cultural competence	6 units	− Participants recognized their biases and privileges. − Participants developed a deeper understanding of others’ experiences. − Participants felt more competent in showing inclusive behavior. − Learning process proved effective.	Qualitative analysis
[47]	2020	Australia	Quantitative	Mental health professionals	38	Nonbinary	1 unit	− Significant increase in perceived knowledge before and after the training. − No change in attitudes. − Increase in confidence in one’s competence.	Self-report (perceived knowledge; attitudes towards the inclusion of transgender women in domestic violence services scale)
[48]	2018	USA	Quantitative	Mental health professional	423	Cultural competence	1 h	− Participants increased their knowledge and expressed a desire for further training. − Self-reports of behavior changes and increased confidence in practice.	Self-report without validation (evaluation of the module)
[49]	2023	Germany	Mixed-methods	Educator	81	Intercultural competence	Many days	− Training was well-rated, especially for professionalization and practicality. − Less effective in migrant-heavy settings, with lower improvements in practice. − Participants who felt interculturally competent showed defensiveness, needing greater trainer attention.	Evaluation questionnaire and interviews
[50]	2018	USA	Quantitative	University staff	108	Diversity	4 weeks	− Online training was practical in fostering cultural competence and learning. − Training increased participants’ appreciation of diversity in education. − Participants gained a greater awareness of social privileges following the training. − Training enhanced willingness to learn about others’ experiences and cultures.	Self-report (value of diversity; awareness of privilege; openness to learning; self-efficacy; geographic background; online communication preference)
[51]	2022	Romania	Quantitative	Mental health professionals	86	LGBTQ	2 days	− Most found the training informative and helpful; nearly all would recommend it. − Reduced explicit/implicit bias and improved LGBTQ-affirmative skills. − No significant differences between face-to-face and online training results.	Self-report (modern homonegativity scale; sexual orientation implicit association test; sexual orientation provider competency scale; gay affirmative practice scale; gay affirmative practice scale; LGBTQ-affirmative practice intentions)

There was no critical appraisal regarding the studies’ sources of evidence, as all studies were considered regardless of quality. This is because the quality of the studies did not constitute the primary focus of this review, as training programs are so individualized and challenging to comparatively evaluate that a review of the studies alone would not add significant value. It was concluded that the programs themselves would need to be assessed to make an informed assessment of the training methods, which proved infeasible. Therefore, the training programs were categorized according to duration, modality (synchronous or asynchronous format), and use of simulations, without further examination of their quality.

A list of the relevant training outcomes is now shown in Table 3.

Four studies employed a mixed-methods design [29, 30, 33, 49]. Only two studies employed a qualitative approach [42, 46], whereas 17 used a quantitative research approach to assess the effectiveness of training [31, 32, 34–41, 43–45, 47, 48, 50, 51]. Fourteen studies were conducted in the United States [31–36, 38, 39, 42–46, 48], two in Germany [29, 49], two in Australia [37, 47], and others in Romania [51], Pakistan [33], South Korea [30], Iran [40], and Israel [41]. Sample sizes varied. The smallest study, of only 11 participants, examined the effects of training quantitatively [44]. The study with the highest number of participants, 3016, also quantitatively assessed the impact of training [31]. Most studies ranged between 39 and 182 participants. A list of the exact number of participants per study is also provided in Table 3.

Sixteen studies addressed cultural competence [29–34, 37, 40–46, 48, 49], six addressed LGBTQ, nonbinary, or gender diversity [31, 36, 38, 45, 47, 51], one addressed diversity in general [50], one addressed disability [39], one addressed prejudice [35], and one addressed belief generation [34]. Only three studies have evaluated the effect of a single training on multiple diversity characteristics [31, 34, 45].

Nine training courses were offered to health professionals [30, 32, 33, 35–37, 39, 41, 45]. Seven studies focused on mental health professionals [29, 38, 43, 44, 47, 48, 51]. Five training courses were provided to teachers or university staff [40, 42, 46, 49, 50], and two to employees [31, 34].

Most studies used asynchronous training designs (14 studies), whereas synchronous designs (six studies) or simulations (three studies) were less frequent. Nine studies examined trainings with a duration of approximately one hour or less, six studies examined trainings with a duration of roughly one working day (ranging from one to eight hours), and eight studies focused on trainings with a duration of more than eight hours. The most common form of training delivered across the included studies was asynchronous training, which typically lasted up to an hour. This is described in greater detail in Table 4 below.

One study examined synchronous one-hour training sessions and reported no significant effects on competence development [45].

In training sessions lasting eight hours or more, effects were observed at all levels of competency. At the affective level, cultural awareness, desire, and encounters increased [40, 42, 43]. At the cognitive level, cultural knowledge was improved [40, 43]. At the behavioral level, cultural skills and transcultural teaching behavior also progressed [40].

For training sessions lasting longer than eight hours, a reduction in LGBTQ prejudice was observed, although no changes in LGBTQ cultural humility were noted [38, 51]. At the cognitive level, knowledge of LGBTQ-affirming cognitive behavioral therapy increased [38]. At the behavioral level, participants improved their LGBTQ-affirming cognitive behavioral therapy skills and behaviors [38, 51]. In a general evaluation, the training was reported to be helpful and meaningful, with no differences found between online and face-to-face formats [51].

Six studies examined asynchronous training lasting less than an hour. On the affective level, some studies reported improvements in cultural awareness and willingness to address racial bias across various encounters [31, 41], whereas others noted no change in attitudes [47]. Cognitive level improvements in knowledge about culture, disability, and nonbinary identities were also reported [39, 47, 48]. At the behavioral level, participants self-reported behavioral changes and improvements in perceived skills [41, 48]. The training was generally judged to be positive [41, 48].

One study reported no effects of asynchronous training, taking between one and eight hours [34]. However, other studies did show an increased awareness of microaggressions and bias [35], and participants demonstrated more inclusive behaviors and improved skills for interacting with minorities [37]. The training tool was stated to be helpful by participants [37].

Several studies also communicated improvements following training sessions lasting longer than eight hours. At the affective level, participants demonstrated increased awareness of diversity, social privilege, and greater openness to new experiences [29, 50]. Cognitive knowledge of prejudice and discrimination generally increased; however, some participants indicated that the training could have benefited from a deeper theoretical foundation [36, 49]. At the behavioral level, self-efficacy and the willingness to confront discrimination increased [50]. Despite overall positive feedback, some participants experienced technical problems, and those who considered themselves to be already interculturally competent reported experiencing defensive reactions [49].

In two studies, a one-hour simulation increased participants’ intercultural competence [34]. At the affective level, negative emotions toward minority groups were reduced, and attitudes improved [35]. No specific effects were reported at the cognitive or behavioral levels.

For simulations lasting more than eight hours, participants demonstrated improved intercultural skills and assessed the training to be useful [33].

Further information on the precise effects across affective, cognitive, and behavioral levels for each study is presented in Table 4.

**Table 4:** Summary of the results from the included studies regarding the training method (synchronous, asynchronous, or simulation) and the duration of training, categorized by cognitive, affective, and behavioral competencies

	One hour or less	Up to eight hours	Days or weeks
**synchronous**	One study with no effects [45]	**Affective** Cultural awareness [40, 42, 43], and cultural encounters [43] increased. **Cognitive** Cultural knowledge increased [40, 43] **Behavioral** Cultural skills and transcultural teaching behavior increased [40] **General Assessment** The training was considered positive [42]	**Affective** Reduction of one’s own LGBTQ prejudices [51], but no effect on LGBTQ cultural humility [38] **Cognitive** LGBTQ-affirmative CBT knowledge increased [38] **Behavioral** LGBTQ-affirmative VBT Skills [38, 51] and behaviors regarding LGBTQ [51] improved. **General Assessment** The training was described as helpful and meaningful. No differences were found between the online and face-to-face versions of the training (51)
**asynchronous**	**Affective** Cultural encounters [31, 32, 41], and the willingness to confront one’s own racial biases [31] improved. In one study, an attitude improvement was measured [41]; in another, it was not [47] **Cognitive** Participants increased their knowledge of culture [41, 48], disability [39], and nonbinary people [47] **Behavioral** Participants reported changes in their behavior [31, 48], an improvement in their own perceived skills [41, 47], and greater perceived security in practice [48] **General Assessment** The training was judged to be helpful and positive [41, 48]	One study with no effects [30] **Affective** Increased awareness of microaggressions, privileges, prejudices, and stereotypes achieved [46] **Cognitive** -- **Behavioral** Following the training, participants demonstrated more inclusive behavior [46] and acquired practical skills in dealing with minorities [37] **General Assessment** An online tool with brief interventions was considered helpful [37]	**Affective** Awareness of diversity was improved [29]. Participants showed increased awareness of social privileges, the value of diversity in educational institutions, and openness and willingness to try new experiences [50] **Cognitive** Participants’ knowledge increased [30, 44]. Participants expressed a desire for more theoretical background knowledge to avoid uncertainties in implementation [49]. Institutions with a large number of employees with a migration background rated the training as less effective and reported less improvement in knowledge and practice [49] **Behavioral** Self-efficacy [36] and the willingness to stand up against prejudice and discrimination increased [50] **General Assessment** A fundamental improvement in cultural competence was achieved [44, 50] In general, the online training received positive feedback [29, 36]. Technical problems posed a challenge [49]. Participants who described themselves as interculturally competent showed defensive behavior toward the training [49]
**simulation**	The intercultural competence of the participants increased [34] **Affective** Training reduced negative emotions toward minority groups and improved attitudinal responses [35] **Cognitive** -- **Behavioral** --		Participants had improved intercultural skills [33] Participants found the training useful [33]

## Discussion

Through the scoping review, we identified 23 studies that address online diversity training published between 2018 and 2024. Our findings indicate that online diversity training is beneficial across different settings. Almost all studies promoted the cognitive, affective, and behavioral levels of competence acquisition. Effects were observed for both synchronous and asynchronous training formats, although some effects were more pronounced in more extended training. No effects were found in three of the 23 studies. This supports the assumption that online diversity training—whether in simulation, synchronous, or asynchronous formats—represents a viable strategy for enhancing diversity competence in the healthcare sector. One caveat is that the effectiveness of training depends on how the training is designed and delivered. Effects were observed across different countries.

Many studies have focused on cultural, intercultural, or transcultural competencies [29, 30, 32–34, 37, 40–46, 48, 49]. Several training courses focused on enhancing participants’ ability to address different cultures in various settings, including care settings [29, 30, 32, 33, 37, 41, 43–45, 48]. Participants reported improved skills in understanding cultural differences and working effectively with people from diverse cultural backgrounds. Many studies also reported improved cultural awareness of and sensitivity to other cultures. In addition, participation in training increased attendees’ willingness to confront their prejudices and the actions resulting from them. However, more intensive training over a more extended period is likely to have a greater impact on this willingness.

A few studies focused on transgender, nonbinary identities, or gender more broadly [31, 36, 38, 45, 47, 51]. These trainings focused on addressing LGBTQ patients appropriately and possible prejudices against LGBTQ patients. Participants reported an increased sense of confidence in dealing with LGBTQ patients, as well as increased knowledge of how to treat them effectively. Again, the online training courses showed great potential for knowledge generation and impact at the affective and behavioral levels.

Few studies focused on broader diverse competencies or included multiple diversity dimensions and their intersectional interactions.

The individual studies and the training courses they evaluated employed a range of training methods. The most considerable differences in modality were found in the forms of synchronous, asynchronous, and simulation training. Synchronous training, which lasted either one day or several weeks, was found to be helpful. Asynchronous training was convenient because it could be flexibly integrated into existing work commitments, but it had mixed results in terms of its effectiveness. In some cases, it led to increased knowledge, while in others, it did not result in behavioral changes. The few simulation-based training courses considered also had positive effects and could supplement other vital training components by recreating real-life scenarios.

The studies included in this scoping review demonstrate that the duration of training has a significant impact on its effectiveness. Training sessions of less than an hour led to fewer behavioral changes but sometimes resulted in improved knowledge. Training sessions lasting between one and eight hours produced mixed results. Some studies reported increased sensitivity to diversity, whereas others reported no significant effects. In contrast, training sessions exceeding eight hours yielded the most substantial improvements among participants, including increased knowledge, a greater willingness to confront prejudice, and measurable behavioral change.

Nevertheless, some studies also identified barriers to effective training delivery. For example, one study noted technical issues that affected participation in online training, including poor internet connections and difficulties with software operation.

Although we did not conduct a formal quality assessment of the included studies, several limitations should be considered. Many studies had relatively small sample sizes, particularly in some quantitative analyses, which may affect the generalizability of results. In addition, self-report bias and social desirability are likely prevalent in many included studies, as most relied on participant self-assessment to measure outcomes. In some cases, very little information or context was provided about the training interventions and how their effectiveness was measured, which limited the interpretability of the findings. Furthermore, by excluding studies that focused primarily on students, the generalizability of our results to early-career professionals is limited.

Another important limitation is the dominance of studies from the United States, which may introduce geographical bias and restrict the applicability of findings to other contexts. Only 23 studies were included over ten years, further limiting the breadth of available evidence.

Additionally, the broad scope and complexity of the search strategy may have led to the inclusion of some irrelevant studies and the unintentional exclusion of relevant ones. The review also excluded gray literature and training programs that have not been formally evaluated in studies, despite the fact that such programs may be highly effective in practice. There was substantial methodological and content-related heterogeneity among the included studies, and the evaluation of training effectiveness was often strongly dependent on the trainers themselves.

It should also be noted that, as a scoping review, our methodology did not include a formal appraisal of study quality, which is a well-known limitation of this review type. However, many studies did provide detailed descriptions of their methods and evaluation techniques, which increased transparency. Many studies utilized established and validated instruments, such as the Clinical Cultural Competence Questionnaire (CCCQ) to measure outcomes. Still, a considerable number of studies relied on self-developed or adapted scales without further validation, which reduces the reliability and comparability of their results. This limitation should be taken into account when evaluating the overall effectiveness of online diversity training.

Overall, these limitations should be considered when interpreting the findings, but the available evidence suggests that online diversity training appears to have a positive effect on participants.

The effect sizes associated with different training durations for specific competency levels remain uncertain. A meta-analysis should systematically extract and analyze individual effect sizes from existing studies to provide clearer insights into their impact. Future studies may also consider the personal training methods used. Examining training content will add greater differentiation, which is not possible from the study descriptions alone.

Our study’s clear strengths lie in its evaluation of various training programs across different groups based on various competence dimensions and in terms of both time and modality. This shows that training programs, given their varying objectives, have the potential to be effective training measures.

Online training methods to improve diversity competence offer several advantages for the healthcare sector, particularly in terms of flexibility and resource efficiency. Asynchronous formats allow participants to learn at their own pace and can reduce costs by minimizing the need for live trainers. Focusing on well-designed and appropriately implemented online diversity training may, therefore, be especially beneficial in settings where time and financial resources are limited. However, this should not be interpreted as a general preference for online over face-to-face formats; when sufficient resources are available, face-to-face formats remain valuable and are not considered inferior. Expanding the availability of high-quality online training could help address existing barriers to participation in healthcare organizations. However, the evidence regarding the effectiveness of online diversity training is mixed. While many studies indicate positive effects, especially in the cognitive and affective domains of diversity competence, several studies reported no effect or only minimal changes in behavioral outcomes. The impact of online diversity training, therefore, appears to be context-dependent, and sustained behavioral change remains limited according to current evidence.

In conclusion, online diversity training holds promise for enhancing cultural competence among healthcare workers, particularly in terms of knowledge and attitudes. Nevertheless, further research is needed to determine the conditions under which these interventions are most effective and to better understand their impact on long-term behavioral change.

## Data Availability

No datasets were generated or analysed during the current study.
